# Redefining communication in mental healthcare: generative AI for neurodivergent equity and non-verbal autistic inclusion

**DOI:** 10.3389/fpsyt.2025.1611101

**Published:** 2025-08-13

**Authors:** Akhil P. Joseph, Anithamol Babu

**Affiliations:** ^1^ School of Social Work, Marian College Kuttikkanam Autonomous, Kuttikkanam, Kerala, India; ^2^ School of Social Work, Tata Institute of Social Sciences Guwahati Off-Campus, Jalukbari, India

**Keywords:** generative AI, non-verbal autism, neurodivergent communication, ethical AI integration, mental healthcare equity

Non-verbal autistic individuals represent one of the most profoundly underserved populations in contemporary mental healthcare, not simply due to service inadequacy, but primarily because dominant communicative norms fundamentally misalign with their embodied and sensory modes of expression (1, 2). Unlike their verbally fluent autistic peers, non-verbal autistic individuals often rely on alternative expressive forms such as echolalia, rhythmic movement, gesture, or sensory-based signalling—modes which are culturally devalued and frequently misclassified in clinical contexts as symptoms of disorder or communicative deficiency (3, 4). Their internal emotional states, affective needs, and attempts at relational communication are frequently interpreted through reductive behavioral lenses or omitted altogether from psychiatric models that prioritize speech, linear reasoning, and neurotypical expressivity (5). These interpretive frameworks contribute to a persistent pattern of diagnostic misrecognition, emotional misattunement, and therapeutic failure. Prevailing models of care, including cognitive-behavioral therapy (CBT), speech-focused interventions, and standardized diagnostic assessments such as the Autism Diagnostic Observation Schedule, Second Edition (ADOS-2), remain heavily dependent on speech-based cues, linear verbal reasoning, and norm-referenced behavioral outputs (6, 7). While these frameworks may be effective for verbally fluent individuals, they are poorly suited for capturing the communicative depth, emotional nuance, and sensory- affective realities of non-verbal autistic individuals.

Despite scattered research on augmentative and alternative communication (AAC) systems, behavioral intervention strategies, and assistive technologies in educational contexts, current scientific inquiry continues to fall short in addressing the affective, relational, and socio- cultural dimensions of non-verbal autistic lives—particularly within psychiatric and psychotherapeutic domains (8–10). These studies tend to prioritize external behavioral outputs over internal emotional states, thereby limiting their capacity to inform practices that are emotionally resonant and neurodivergently aligned. Moreover, few existing models meaningfully incorporate lived experience, embodied knowledge, or the sociopolitical context in which non-verbal autistic individuals interface with mental healthcare systems (11, 12). This lack of representational justice reveals a deep theoretical and practical gap that cannot be resolved through technological enhancement alone—it requires a paradigmatic shift in how communication, personhood, and therapeutic alliance are conceptualized. Unless research and clinical paradigms are expanded to legitimize and respond to non-verbal modes of expression, non-verbal autistic individuals will remain excluded from both the epistemological foundations and practical realities of equitable mental healthcare.

Generative artificial intelligence (AI) presents a timely and transformative intervention to reconfigure the communicative foundations and relational dynamics of mental healthcare by addressing the expressive gaps that persistently marginalize non-verbal autistic individuals. Unlike conventional tools that prioritize speech interpretation, generative AI systems— particularly those integrating natural language processing and multimodal affective recognition—can process complex, context-rich inputs such as facial micro-expressions, vocal tones, gaze patterns, repetitive movement, autonomic responses, and environmental cues, thereby generating outputs that translate non-verbal emotional states into coherent, relationally relevant messages (13, 14). This is not about reducing neurodivergent communication into neurotypical language; rather, it is about affirming its validity and rendering it legible within therapeutic frameworks. Current AI- augmented systems like Affectiva’s emotion analytics, Cognoa’s pediatric behavioral health platforms, and increasingly adaptive AAC tools suggest early but promising directions in this regard (15, 16). For example, in overstimulating environments such as classrooms or clinics, AI-enabled wearables could detect physiological and behavioral stress markers and generate real-time alerts like “I feel overwhelmed and need a break,” allowing clinicians or educators to respond promptly and empathetically. These tools can de- escalate distress, reduce misinterpretation, and promote communicative trust. While preliminary evidence supports the use of AI in ASD-related contexts, empirical research specifically examining generative AI applications for non-verbal autistic individuals remains limited but emergent, warranting focused scholarly attention and investment. However, it is crucial to emphasize that generative AI cannot and should not be expected to replace human empathy, clinical judgment, or the relational nuance of therapeutic alliance. It cannot fully interpret subjective experience, guarantee cultural sensitivity without intentional design, or autonomously validate emotional truths. Rather than a panacea or autonomous agent of care, generative AI must be positioned as a relational co-participant—an interpretive scaffold that supports, but never substitutes, the human work of listening, validating, and building trust. Recognizing its limits is essential to avoiding technological idealism and ensuring that its integration enhances rather than distorts the therapeutic process.

The adaptive capacity of generative AI offers transformative potential to reconfigure how mental healthcare systems engage with the diverse communicative realities of non-verbal autistic individuals—across developmental stages, intersecting identities, and care contexts— by translating nuanced, embodied expressions into therapeutic insight without collapsing their meaning into neurotypical norms (17). From preverbal toddlers requiring early detection of sensory distress and communication delays, to school-age children needing support in emotion identification and social interaction, AI-integrated platforms can offer real-time feedback, visual prompts, and personalized engagement in classrooms and therapeutic environments (18). For adolescents managing developmental shifts, peer dynamics, and emerging self-concepts, generative AI could enable safe spaces for emotional journaling, avatar-based role-play, or voice-assisted storytelling—tools that empower identity exploration while mitigating communicative alienation. For adults, especially those at the intersections of autism, intellectual disability, trauma, and gender nonconformity, AI can provide continuous affective tracking, adaptive stress response strategies, and relational continuity in both institutional and home-based care (19).

Equally important is the capacity of generative AI to attend to the sociopolitical dimensions of communication by embedding intersectional insights into its interpretive models (20). Whether shaped by language barriers, racialized diagnostic disparities, class- based access limitations, or culturally situated expressions of affect, AI tools must be designed to engage with these contextual nuances, thereby avoiding the homogenization of neurodivergent expression. When attuned to these factors, generative AI can function as both an interpretive aid and a structural corrector, disrupting entrenched speech-dominant paradigms that constrain therapeutic engagement and marginalize non-verbal communicative forms. Its strength lies in its adaptability: systems can evolve in response to user-specific patterns, culturally embedded meanings, and age-appropriate forms of expression, rendering care more responsive, ethical, and inclusive (21). To frame generative AI merely as an assistive communication tool is to overlook its revolutionary potential as a platform for epistemic justice, relational equity, and clinical transformation across the lifespan.

The development, deployment, and validation of AI tools within mental healthcare must be guided by the lived expertise of neurodivergent communities, supported by interdisciplinary collaboration across clinical, technological, and social domains. Participatory design processes must actively involve autistic individuals, caregivers, AAC users, and clinicians to co-create systems that reflect real-world communicative practices and emotional priorities. Pilot trials must go beyond traditional efficacy metrics to capture subjective measures such as emotional resonance, perceived respect, empowerment, and trust in therapeutic relationships. Research methodologies should include multimodal validation strategies that combine quantitative metrics—such as physiological biomarkers and behavioral indicators—with qualitative ethnographies, narrative interviews, and community-led data interpretation. Crucially, AI systems must be trained to recognize affect not as a decontextualized output but as a relational, ecologically situated signal shaped by personal history, environment, and cultural norms. Furthermore, AI-generated insights must be treated not as definitive clinical verdicts but as provisional and dialogic prompts that invite further interpretation, discussion, and negotiation.

While the ethical potential of generative AI is foregrounded throughout this discourse, its deployment also raises profound risks, including the normalization of surveillance, clinical coercion, algorithmic misjudgment, and the commodification of affective data. These threats are especially acute in under-resourced or overly institutionalized environments, where AI may reinforce systemic hierarchies rather than subvert them. To ensure equitable integration, a phased, justice-oriented implementation strategy must guide its development. This must include a clear delineation of potential harms such as data misuse, behavioral over-surveillance, and the uncritical pathologization of AI-interpreted signals. Safeguards—such as algorithmic auditing, data minimization, neurodivergent oversight, and real-time interpretive feedback loops—must be implemented to counterbalance these risks and uphold ethical integrity. In Phase One, participatory co-design with non-verbal autistic individuals, caregivers, and neurodivergent clinicians is essential to produce culturally grounded, developmentally responsive prototypes. Ethical safeguards—such as dynamic, ongoing consent mechanisms, transparent data governance, and community-led oversight boards—must be embedded from inception, particularly across both developed and developing countries. In Phase Two, these systems should undergo multi-site testing in classrooms, clinics, and community settings using mixed- method designs that assess clinical accuracy, emotional attunement, and relational trust. Biometric analytics, caregiver feedback, and co-produced narrative insights should together evaluate AI’s therapeutic validity. Phase Three demands the translation of successful models into sustainable infrastructure. In high-income countries, this requires AI literacy training, algorithm auditing, and embedding AI insights within interdisciplinary teams to prevent overreliance or diagnostic substitution. In low-resource contexts, mobile-based, open-source platforms co-designed with local stakeholders can bridge access gaps—provided they avoid dependency on foreign-trained models that overlook local expressions of distress or relationality. Globally, open data initiatives, multilingual AI frameworks, and cross-sectoral regulatory agreements must ensure scalability without cultural erasure. For this to occur, institutions must actively disinvest from speech-centric models of therapeutic engagement and reorient their clinical philosophies toward principles of epistemic humility, pluralism, and structural justice. The implementation of generative AI must be accompanied by a justice- oriented research agenda that is interdisciplinary in nature, drawing from affective computing, neuroethics, critical disability studies, participatory design, and mental health implementation science.

To sustain this transformation, structural changes across the mental healthcare ecosystem are imperative and must occur across multiple, interdependent domains. Clinical protocols and diagnostic guidelines must explicitly recognize non-verbal and embodied communication as valid, affectively rich, and diagnostically meaningful, and these recognitions must be operationalized in clinical assessments, training curricula, and institutional standards. Funding bodies and innovation agencies must prioritize inclusive, co-produced technological development, ensuring sustained support for community-based pilot projects, multi-site implementation research, and neurodivergent-led evaluation processes over commercial scalability or investor-driven acceleration. Editorial boards, academic journals, and clinical training institutions must challenge exclusionary epistemologies that devalue experiential knowledge and must elevate neurodivergent authorship, peer review, and editorial leadership. National regulatory bodies should issue ethical guidelines specific to AI-mediated communication in psychiatric and psychological care, while professional associations must establish continuing education standards that include neurodiversity-informed AI literacy. Accreditation bodies should integrate participatory design and ethical AI governance as core benchmarks for evaluating mental healthcare programs and innovations. Public institutions and data consortia must develop open-access, culturally grounded, and community-curated datasets that actively counter the representational biases embedded in conventional training corpora. Ethical AI development requires dynamic, relational consent; data sovereignty; enforceable accountability; culturally relevant interpretation frameworks; and community-driven oversight mechanisms that are ongoing, accessible, and enforceable. These are not enhancements but non-negotiable imperatives. Generative AI will only democratize mental healthcare if it is governed by, accountable to, and continuously shaped by the very individuals and communities it seeks to represent—otherwise, it risks becoming a polished instrument of systemic exclusion, epistemic violence, and clinical erasure.

Therefore, mental healthcare must confront its epistemic blind spots by urgently investing in participatory, neurodivergent-led, transdisciplinary research that redefines communicative justice through inclusive, ethically grounded, and empirically validated generative AI interventions with truly global applicability—particularly for non-verbal autistic individuals. This research must directly engage with complex and unresolved questions: How can informed consent be meaningfully operationalized when communication is technologically mediated, embodied, and non-verbal? What methodological strategies ensure ecological and cultural validity when interpreting multimodal affective data across linguistically diverse, socioeconomically stratified, and geopolitically varied healthcare systems? What safeguards are needed to prevent algorithmic outputs from reinforcing diagnostic biases, intensifying global health inequities, or flattening culturally specific communicative practices? These questions are especially urgent for non-verbal autistic populations who remain excluded from dominant speech-centered diagnostic frameworks and therapeutic paradigms. Addressing these challenges requires robust integration of affective computing, human-computer interaction, disability studies, neuroethics, communication theories, implementation science, and lived experiences research across regional, linguistic, and cultural contexts. Developing multi-phase longitudinal studies, AI-assisted therapeutic prototypes grounded in ethnographic insight, and comparative global policy frameworks is critical not only for building an internationally credible foundation for equitable AI deployment, but also for ensuring that non-verbal autistic individuals are no longer treated as invisible subjects within mental healthcare systems. This effort represents a research and justice mandate—one that challenges dominant epistemologies while advancing neurodivergent expression within a culturally responsive and ethically grounded mental healthcare paradigm.
